# Fecal Microbiota Transplantation Reduces *Campylobacter jejuni* Colonization in Young Broiler Chickens Challenged by Oral Gavage but Not by Seeder Birds

**DOI:** 10.3390/antibiotics12101503

**Published:** 2023-10-02

**Authors:** Jinji Pang, Ashenafi Feyisa Beyi, Torey Looft, Qijing Zhang, Orhan Sahin

**Affiliations:** 1 Department of Veterinary Microbiology and Preventive Medicine, College of Veterinary Medicine, Iowa State University, Ames, IA 50011, USA; pjj0702@iastate.edu (J.P.); afbeyi@iastate.edu (A.F.B.); zhang123@iastate.edu (Q.Z.); 2 Department of Statistics, Iowa State University, Ames, IA 50011, USA; 3 National Animal Disease Center, United States Department of Agriculture, Ames, IA 50010, USA; torey.looft@usda.gov; 4 Department of Veterinary Diagnostic and Production Animal Medicine, College of Veterinary Medicine, Iowa State University, Ames, IA 50011, USA

**Keywords:** *Campylobacter*, fecal microbiota transplantation (FMT), broilers, gut microbiota, direct infection model, seeder bird infection model

## Abstract

*Campylobacter* spp., particularly *C. jejuni* and *C. coli*, are major food safety concerns, transmitted to humans mainly via contaminated poultry meat. In a previous study, we found that some commercial broiler farms consistently produced *Campylobacter*-free flocks while others consistently reared *Campylobacter*-colonized flocks, and significant differences in the gut microbiota compositions between the two types of farm categories were revealed. Therefore, we hypothesized that gut microbiota influences *Campylobacter* colonization in poultry and that the microbiota from *Campylobacter*-free flocks may confer colonization resistance to *Campylobacter* in the chicken intestine. In this study, two fecal microbiota transplantation (FMT) trials were performed to test the hypothesis. Newly hatched chicks were given FMT via oral gavage of the cecal content of *Campylobacter*-free adult chickens (treatment groups) or PBS (control groups) before the feed consumption. Approximately two weeks after the FMT, the birds were challenged with *C. jejuni* either by oral gavage (trial 1) or by co-mingling with *Campylobacter*-colonized seeder birds (trial 2) to evaluate the potential protective effect of the FMT. Cecal contents were collected (3 times, 5 days apart) to determine the *Campylobacter* colonization levels via culture and microbiota compositions via 16S rRNA gene sequencing. FMT reduced cecal *Campylobacter* colonization significantly (log_10_ 1.2–2.54 CFU/g) in trial 1 but not in trial 2, although FMT significantly impacted the diversity and compositions of the gut microbiota in both trials. Several genera, such as *Butyricimonas*, *Parabacteroides*, *Parasutterella*, *Bilophila*, *Fournierella*, *Phascolarctobacterium*, and *Helicobacter*, had increased abundance in the FMT-treated groups in both trials. Furthermore, *Campylobacter* abundance was found to be negatively correlated with the *Escherichia* and *Ruminococcus_torques_group* genera. These findings indicate that even though FMT with adult cecal microbiota can positively affect the subsequent development of the gut microbiota in young broilers, its inhibitory effect on *Campylobacter* colonization varies and appears to be influenced by the challenge models.

## 1. Introduction

*Campylobacter*, a zoonotic and foodborne bacterial pathogen, is a major cause of gastroenteritis and diarrhea in humans worldwide [1]. According to the most recent Centers for Disease Control and Prevention (CDC) FoodNet surveillance report, the foodborne infection incidence was highest for *Campylobacter* (19.2 cases per 100, 000 population) among the laboratory-confirmed bacterial and parasitic illnesses at 10 sites in 2022 in the United States [2]. Among all the pathogenic *Campylobacter* species, *C. jejuni* is reported to be the main cause of human campylobacteriosis [3]. Diarrhea, fever, and stomach cramps are common symptoms of *C. jejuni* infection [4]. More severe symptoms, such as acute appendicitis, ulcerative colitis, and Guillain–Barré syndrome, can also occur less frequently [5]. Most *Campylobacter* infections in humans are sporadic and predominantly associated with the poor handling of contaminated raw chicken meat and consumption of undercooked chicken [6,7,8]. Commercial poultry, including chickens and turkeys, is frequently colonized with *Campylobacter* in the intestinal tract without showing any significant clinical or pathological signs of infection in natural settings [9,10,11,12,13]. Studies conducted around the world have reported a high but variable flock/farm level prevalence, ranging from 2% to 100% in various types of production systems [14,15,16,17,18,19,20,21]. Typically, the prevalence of *Campylobacter* increases as the birds grow and reach the highest points at the slaughter age for broiler chickens [20]. As such, chicken carcass is frequently contaminated with *Campylobacter* during slaughter and can subsequently transmit the organism to humans via multiple routes [22,23]. 

Preharvest control of *Campylobacter* in poultry production is a critical step in order to reduce carcass contaminations during processing and subsequent foodborne transmission [24,25]. However, poultry houses can be contaminated by *Campylobacter* in many different ways, making controlling *Campylobacter* in poultry a very challenging task [20]. Although biosecurity-based interventions can have a noticeable effect on lowering the overall flock prevalence on farms, they do not always have a consistent and predictable effect on controlling *Campylobacter,* and their effectiveness in controlling flock prevalence is difficult to assess in commercial settings [20,23,26]. Multiple non-biosecurity measures (e.g., vaccines, bacteriocins, phages, feed additives, competitive exclusion products, etc.) have been evaluated to control *Campylobacter* in live birds with various outcomes [20,24,25,27,28]. Significant reductions in *Campylobacter* colonization in chickens were observed in several studies involving bacteriophages, various types of vaccines, and feed additives (29, 30, 31, 32, 33). 

In contrast to these promising results, many other studies found no significant effect of a wide range of preharvest intervention approaches on *Campylobacter* colonization in live birds. These include studies on plant-derived compounds, probiotics, and numerous types of vaccines (34, 35, 36, 37, 38). Collectively, the findings from the multiple investigations described above clearly indicate the large discrepancies in the effectiveness of different non-biosecurity-based preharvest intervention methods on *Campylobacter* colonization in chickens and thus point out the need for additional research in order to develop successful and efficient mitigation strategies against *Campylobacter* in poultry.

One such strategy that has become relatively popular and commonly used in recent years for the control of enteric bacteria in food animals revolves around the concept of host microbiota and its perceived role in health and disease. Gut microbiota are a collection of bacteria, archaea, viruses, and fungi found within the gastrointestinal (GI) tract [29]. As a rich microbial community, gut microbiota are important for the overall host health in providing essential nutrients, assisting alimentation, and conferring colonization resistance against pathogens [30]. Manipulation of poultry gut microbiota using dietary products, such as probiotics and prebiotics, has shown promising results in enhancing gut health and food safety [31,32]. It is generally accepted that intestinal microbiota enhances colonization resistance to enteric pathogens by direct and indirect mechanisms, such as competing for nutrients and attachment sites, producing antimicrobial substances, and stimulating an immune response [33,34]. Several commensal members of gut microbiota, such as *Lactobacillus*, *Bacillus*, *Enterococcus*, and *Bifidobacterium*, have been shown to have an inhibitory effect on *Campylobacter* colonization in broilers [35]. Multiple *Lactobacillus* species, including *L. salivarius*, *L. reuteri*, *L. plantarum*, and *L. agilis*, were reported to have the ability to produce bacteriocins or organic acids and significantly reduced *Campylobacter* colonization in broilers under experimental settings [32,35,36]. Likewise, *Bacillus subtilis* PS-216 was shown to have potent anti-*Campylobacter* activity in vitro and significantly lowered *Campylobacter* colonization in the ceca of broiler chickens when continuously supplemented in their drinking water in a recent experiment [37]. Collectively, these studies strongly suggest that a microbiota-based intervention may be a feasible strategy for preharvest control of *Campylobacter* in poultry. One such approach that emerged during the past decades has been the delivery of total microbial communities from healthy adult chickens to newly hatched chicks via a method commonly dubbed fecal microbiota transplantation (FMT). Inoculating young broilers with fecal microbiota transplants from highly feed-efficient donors was shown to affect the cecal microbiota composition and the host’s intestinal development [38]. Moreover, various degrees of success have been observed in the control of enteric foodborne bacteria (e.g., *Salmonella* and *Campylobacter*) in the poultry intestinal tract among different studies employing FMT [24,39,40,41,42,43,44]. 

In a recent study, we showed that there were significant differences in the microbiota composition in the intestines of adult chickens between *Campylobacter*-colonized and *Campylobacter*-free commercial broiler flocks over multiple production cycles [45]. Based on this particular observation and the information available in the published literature, we hypothesized that transplantation of the gut microbiota of *Campylobacter*-free adult chickens to newly hatched chicks could decrease *Campylobacter* colonization in the intestine of recipient birds. To test this hypothesis, cecal contents from the *Campylobacter*-free adult commercial broilers were used as FMT material for newly hatched broiler chicks, which were subsequently infected with *C. jejuni* by either direct oral inoculation or co-mingling with *Campylobacter*-colonized seeder birds. Thereafter, cecal contents were collected periodically at necropsy for quantitative *Campylobacter* culturing and 16S rRNA gene-based microbiota analysis.

## 2. Results

### 2.1. FMT Significantly Lowered C. jejuni Colonization in Broilers Challenged by Oral Gavage but Not via the Seeder Bird Infection Model

The impact of FMT from *Campylobacter*-free adult (5-week-old) commercial broilers to newly hatched broiler chicks on the cecal microbiota configuration and *Campylobacter* colonization in young broiler chicks were assessed in two separate trials. The fecal microbiota transplantations were performed when the chicks were 1-day-old, and the *C. jejuni* challenges were performed approximately two weeks later via either a direct infection model (oral gavage of all individual birds, FMT trial 1) or an indirect infection model (co-mingling with *Campylobacter*-colonized seeder birds, FMT trial 2). In both trials, a subset of chicks was euthanized and necropsied at three designated sampling points following the *C. jejuni* infection (days 5, 10, and 15), and the cecal contents were obtained for the purpose of quantifying the *C. jejuni* loads and analyzing the microbiota composition. 

**Figure 1:**
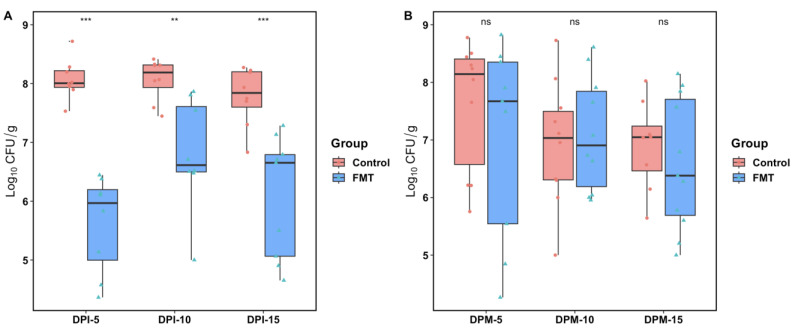
Colonization levels of *C. jejuni* in the cecal contents of broiler chickens following direct infection (FMT trial 1, (**A**)) or seeder bird infection (FMT trial 2, (**B**)) models. Each dot represents the log_10_ transformed CFU/g feces from an individual chicken. The mean values (for 8 birds in each group/DPI in panel (**A**) and 10 birds for each group/DPM in panel (**B**)) are shown as the middle horizontal lines, and the vertical lines stretching out from the means represent the 95% confidence intervals (CI). The red dots and blue triangles on the plots represent individual sample’s log_10_ transformed CFU data from the control and the FMT groups. DPI, day post inoculation with *C. jejuni*; DPM, day post mingling after the introduction of the seeder birds colonized with *C. jejuni*. The *p*-values were calculated using the non-parametric Wilcoxon test (*** *p*-value < 0.001, ** *p*-value < 0.01; ns: not significant).

In FMT trial 1, compared with the control group (given PBS), birds in the FMT group had significant reductions in the cecal *C. jejuni* loads at DPI-5 (*p*-value < 0.0001), DPI-10 (*p*-value < 0.01), and DPI-15 (*p*-value < 0.0001) by the Wilcoxon rank sum test (Figure 1A). The means CFU/g feces in the FMT group were reduced by log_10_ 2.54, 1.2, and 1.7 compared with those in the control group at DPI-5, DPI-10, and DPI-15, respectively. The *C. jejuni* loads were also shown to change significantly over time in the FMT group by the Kruskal–Wallis rank sum test (*p*-value = 0.035), with DPI-5 having the lowest CFU (mean log_10_ 5.62/g) and DPI-10 having the highest CFU (mean log_10_ 6.80/g) (Figure 1A). No such variation in *C. jejuni* loads over time was observed in the control group (*p*-value = 0.259). 

In FMT trial 2, all the seeder birds became colonized by *C. jejuni* after the oral challenge and remained at comparably high colonization levels in both groups throughout the experiment, as determined by culturing cloacal swabs. In contrast to the results from FMT trial 1, the *C. jejuni* colonization levels in the co-mingled birds of the FMT group did not differ significantly from those in the control group for any of the sampling points performed at DPM-5, DPM-10, and DPM-15 (Figure 1B). Even though the means CFU/g feces were reduced by log_10_ 0.57 and 0.32 at DPM-5 and DPM-15 in the FMT group compared with the control group, these differences were not significant (*p*-value = 0.389 and 0.516, respectively). The *C. jejuni* colonization levels did not show any significant changes over time in either the control group or the FMT group, as measured at DPM-5, DPM-10, and DPM-15 by the Kruskal–Wallis rank sum test (*p*-value = 0.574 for the FMT group and *p*-value = 0.216 for the control group).

### 2.2. Microbiota Analysis

#### 2.2.1. 16S rRNA Gene Sequencing Outputs Overview

The cecal microbiota compositions of the birds euthanized at 0, 5, 10, and 15 days following the *C. jejuni* infection in both FMT trials were determined via 16S rRNA gene-based metagenomics. After quality filtering and the chimeric reads removal step, the average reads per sample were 80,214 for FMT trial 1 and 51,655 for FMT trial 2. Rarefaction curves were generated for various alpha diversity metrics (including the observed features, Shannon, Pielou’s evenness, and Faith’s pd) with a depth of 80,214 for FMT trial 1 and 40,000 for FMT trial 2; the results showed that both trials had enough sequencing depth to detect low-abundant bacterial taxa. After filtering out rare amplicon sequence variants (ASVs), 977 ASVs were identified in FMT trial 1 and 1106 ASVs were identified in FMT trial 2 based on a >99% sequence reads similarity. 

#### 2.2.2. FMT Increased Cecal Microbial Species Richness and Phylogenetic Diversity in Young Broilers

To compare the within-sample microbiota diversity of samples between the FMT treatment groups and control groups (received reduced PBS) in both FMT trials, alpha diversity analysis was conducted using the 16S rRNA gene sequencing results on the QIIME 2 platform. Of note, both observed features (ASVs) and the Shannon diversity index were used as measures of predicted species richness (the number of different species present in a community), while both the Pielou’s and Shannon indexes were used as indicators of species evenness (the distribution of relative abundances of individual species present in a community). In addition, Faith’s phylogenetic diversity (Faith’s pd) was used to measure not only the number of species but also the community evolutionary distance within the samples.

**Figure 2:**
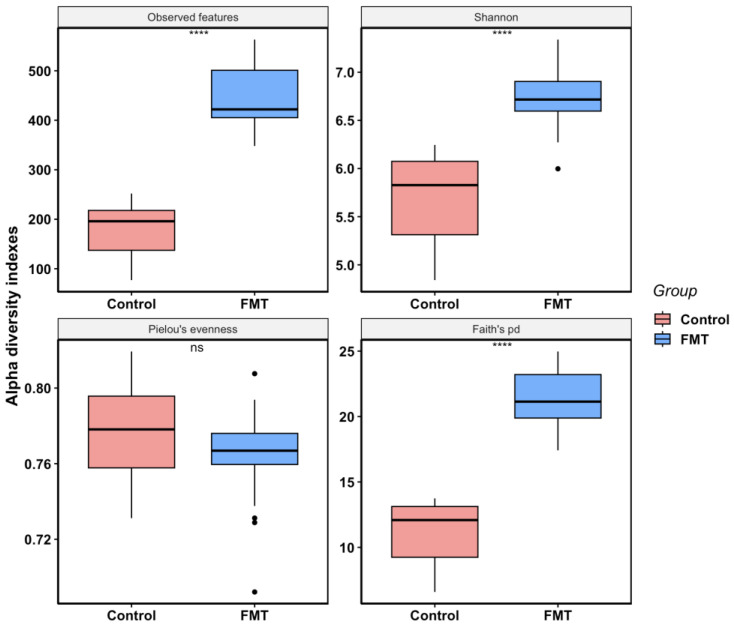
Box plots for alpha diversity metrics (observed features, Shannon, Pielou’s evenness, and Faith’s pd) comparing FMT and control samples in FMT trial 1. Each box shows the combined data from the entire experiment within each group. Horizontal bars inside boxes mark the mid-point of the data. The upper and lower vertical bars represent data values outside the middle 50%. The *p*-values were calculated using a non-parametric Wilcoxon test (**** *p*-value < 0.0001; ns: not significant).

**Figure 3:**
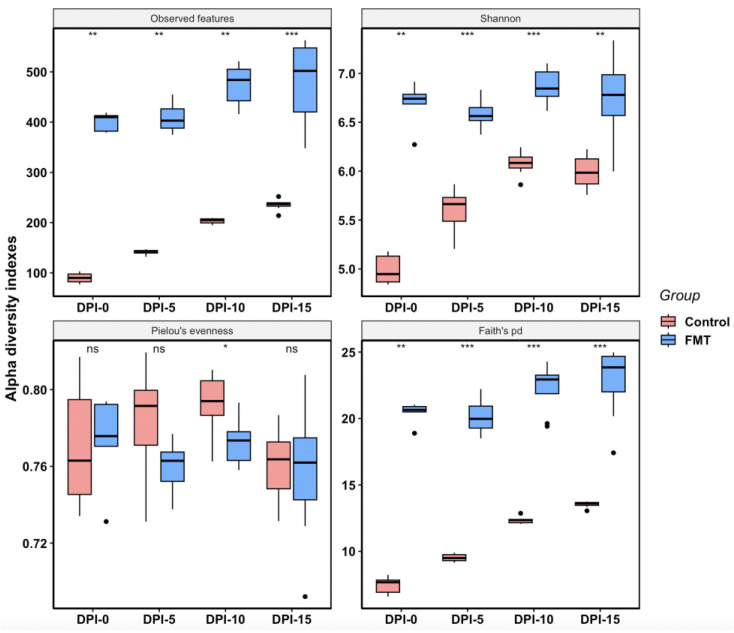
Box plots for alpha diversity metrics (observed features, Shannon, Pielou’s evenness, and Faith’s pd) comparing FMT and control samples from DPI-0, DPI-5, DPI-10, and DPI-15 in FMT trial 1. Each box shows the combined data from a particular sampling event (DPI, day post inoculation) within each group. Horizontal bars inside boxes mark the mid-point of the data. The upper and lower vertical bars represent data values outside the middle 50%. The *p*-values were calculated using the non-parametric Kruskal–Wallis test (*** *p*-value < 0.001, ** *p*-value < 0.01, * *p*-value < 0.05; ns: not significant).

In FMT trial 1, the overall alpha diversity of the FMT group (mean observed features = 446; mean Shannon index = 6.73; mean Pielou’s evenness = 0.77; mean Faith’s pd = 21.57) was much higher than that of the control group (mean observed features = 172; mean Shannon index = 5.70; mean Pielou’s evenness = 0.78; mean Faith’s pd = 10.93) as determined by the majority of diversity metrics analyses (Figure 2). The FMT group not only had a greater number of observed features present but was also more phylogenetically diverse according to Faith’s pd index. Although the FMT group had a significantly higher Shannon index than the control group, indicating higher species richness and evenness in the FMT group, there was no significant difference in the evenness between the two groups, as shown by the Pielou’s evenness index (*p*-value = 0.1316), which only measures the species evenness of the community regardless of the species richness. When the same data were analyzed by the sampling time, the differences between the two groups became more noticeable (Figure 3). At all sampling points, the FMT group had significantly high indexes in the observed features than the control group (mean values of 400 vs. 90 at DPI-0; 408 vs. 14 at DPI-5; 473 vs. 203 at DPI-10; 481 vs. 235 at DPI-15), Shannon (mean values of 6.68 vs. 4.99 at DPI-0; 6.59 vs. 5.60 at DPI-5; 6.87 vs. 6.08 at DPI-10; 6.74 vs. 5.99 at DPI-15), and Faith’s pd (mean values of 20.39 vs. 7.45 at DPI-0; 20.16 vs. 9.52 at DPI-5; 22.33 vs. 12.34 at DPI-10; 22.79 vs. 13.53 at DPI-15), indicating that FMT increased the microbiota species richness and phylogenetic diversity (Figure 3). There was a noticeable and steady increase in species richness and phylogenetic diversity over time as the experiment progressed in the control group; however, such changes in the FMT group were less obvious and moderate only (Figure 3). Except for DPI-10, there were no significant differences in the species evenness between the groups (mean Pielou’s evenness 0.77 vs. 0.77 at DPI-0; 0.76 vs. 0.78 at DPI-5; 0.77 vs. 0.79 at DPI-10; 0.76 vs. 0.76 DPI-15 for FMT vs. control groups, respectively) (Figure 3). 

**Figure 4:**
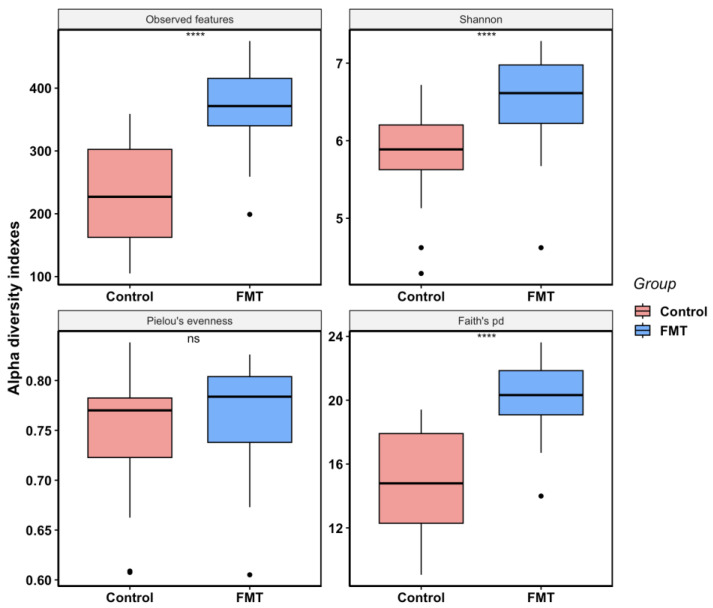
Box plots for alpha diversity metrics (observed features, Shannon, Pielou’s evenness, and Faith’s pd) comparing FMT and control samples in FMT trial 2. Each box shows the combined data from the entire experiment within each group. Horizontal bars inside boxes mark the mid-point of the data. The upper and lower vertical bars represent data values outside the middle 50%. The *p*-values were calculated using a non-parametric Wilcoxon test (**** *p*-value < 0.0001; ns: not significant).

**Figure 5:**
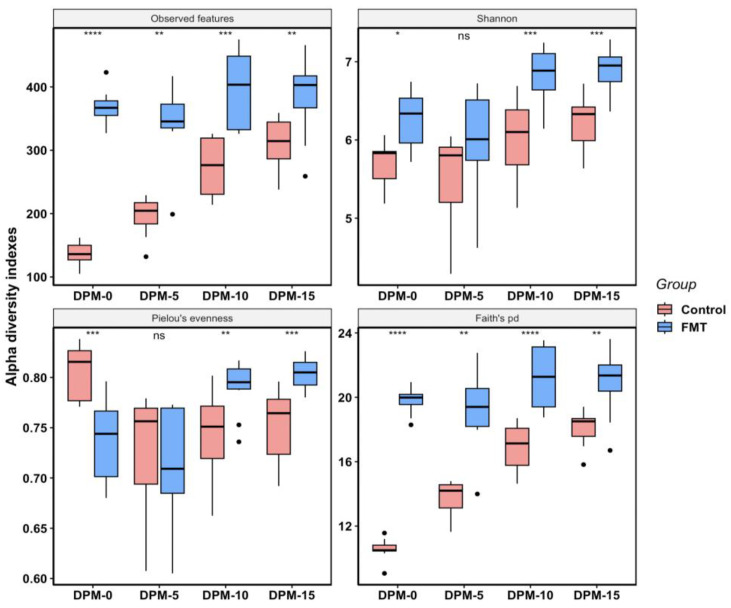
Box plots for alpha diversity metrics (observed features, Shannon, Pielou’s evenness, and Faith’s pd) comparing FMT and control samples from DPM-0, DPM-5, DPM-10, and DPM-15 of FMT trial 2. Each box shows the combined data from a particular sampling event (DPM, day post mingling) within each group. Horizontal bars inside boxes mark the mid-point of the data. The upper and lower vertical bars represent data values outside the middle 50%. The *p*-values were calculated using the non-parametric Kruskal–Wallis test (**** *p*-value < 0.0001, *** *p*-value < 0.001, ** *p*-value < 0.01, * *p*-value < 0.05; ns: not significant).

In FMT trial 2, the overall alpha diversity differences and the dynamics changes between the FMT group (mean observed features = 374; mean Shannon index = 6.53; mean Pielou’s evenness = 0.76; mean Faith’s pd = 20.35) and the control group (mean observed features = 231; mean Shannon index = 5.86; mean Pielou’s evenness = 0.75; mean Faith’s pd = 14.94) were very similar to what was observed in FMT trial 1. FMT increased the microbiota species richness, phylogenetic diversity, and Shannon index but not Pielou’s evenness significantly (Figure 4). As the experiment progressed, there was a clear increasing trend of species richness and phylogenetic diversity in the control group; however, such changes were less prominent in the FMT group (Figure 5). Even though the control group had higher Pielou’s evenness than the FMT group at DPM-0, the reverse was observed during the last two sampling points of the study (mean values of 0.73 vs. 0.81 at DPM-0; 0.79 vs. 0.74 at DPM-10; 0.80 vs. 0.75 at DPM-15 in the FMT and control groups, respectively) (Figure 5).

#### 2.2.3. FMT Resulted in Significant Dissimilarity in Cecal Microbiome Composition as Revealed by Beta Diversity Analysis

**Figure 6:**
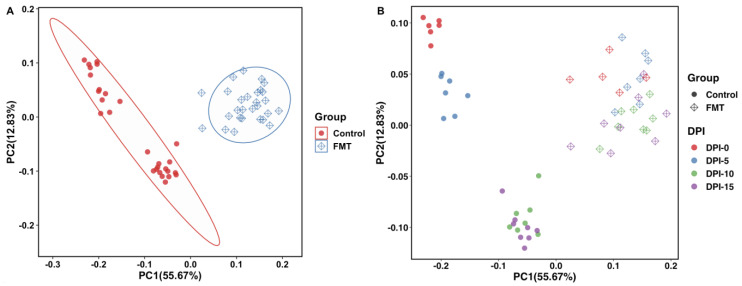
Principal coordinate analysis (PCoA) plots based on weighted UniFrac distances for FMT trial 1. The FMT and control populations were grouped by treatment status (**A**) or by treatment status and sampling times (**B**). The PC1 axis depicts 55.67% of the total variance, and the PC2 axis shows 12.83% of the total variance. Each dot represents microbiota composition in a single sample. Each ellipse was drawn at a 95% confidence level to include most samples in either group (**A**). DPI: day post inoculation.

**Figure 7:**
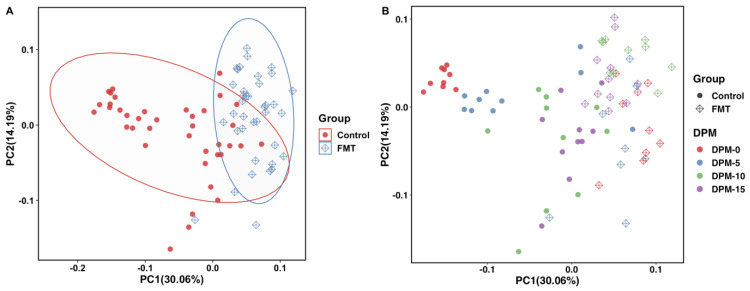
Principal coordinate analysis (PCoA) plot based on weighted UniFrac distances for FMT trial 2. The FMT and control populations were grouped by treatment status (**A**) or by treatment status and sampling times (**B**). The PC1 axis depicts 30.06% of the total variance, and the PC2 axis shows 14.19% of the total variance. Each dot represents microbiota composition in a single sample. Each ellipse was drawn at a 95% confidence level to include most samples in either group (**A**). DPM: day post mingling.

To compare the microbial communities and structures of the samples from the FMT group and the control group for each FMT trial, beta diversity analysis was conducted using the 16S rRNA sequencing results on the QIIME 2 platform. Weighted UniFrac distances (a measure of community dissimilarity that incorporates phylogenetic relationships between the features) were determined and illustrated in a PCoA plot. The two-dimensional visualizations of the weighted UniFrac distance results showed that the overall microbial populations of the FMT groups and the control groups separated clearly from each other and formed two clusters in both FMT trial 1 (Figure 6A) and trial 2 (Figure 7A). When the same data were analyzed by the sampling time, quite similar separation patterns were observed, even though the formed clusters were not tight (Figure 6B and Figure 7B). 

**Figure 8:**
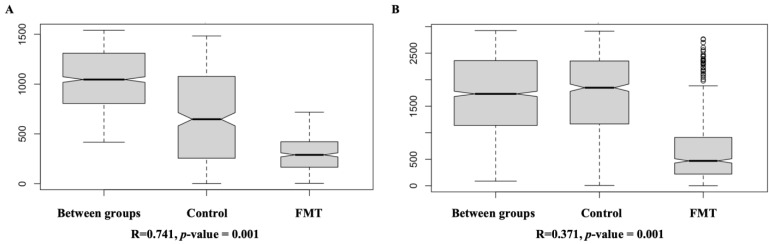
Box plots for the analysis of similarity (ANOSIM) of the cecal microbiota compositions in FMT trial 1 (**A**) and FMT trial 2 (**B**). The analysis was conducted using a Bray–Curtis dissimilarity index based on the amplicon sequence variant (ASV) composition of the total samples within each unit shown on the *x*-axis. The dissimilarity rank values, calculated based on 999 permutations between and within groups, are shown on the *y*-axis. In both figures, “Between groups” indicates the compositional dissimilarities of cecal microbiota between the FMT group and the control group; “Control” indicates the compositional dissimilarities of cecal microbiota within the control group; “FMT” indicates the compositional dissimilarities of cecal microbiota within the FMT group. The round dots on the box plots indicate outliers of permutation test results. The test result is represented by an R-value and the test significance value (*p*-value). An R-value close to “1” suggests strong dissimilarity between groups, while an R-value close to “0” suggests less dissimilarity between groups. A *p*-value less than 0.05 is generally considered statistically significant.

ANOSIM was also conducted to confirm the results from the beta diversity analysis described above. For FMT trial 1, the overall microbiota structure was significantly different between the FMT group and the control group (R = 0.741, *p*-value = 0.001) (Figure 8). In addition, the dissimilarity of the cecal microbiomes between the two groups was much greater than the dissimilarity within each group (Figure 8A). For FMT trial 2, even though the dissimilarity between groups was still significantly higher than that within the FMT group (R = 0.371, *p*-value = 0.001), it was at a similar level to that within the control group (Figure 8B). ANOSIM tests further revealed that the dissimilarity in the gut microbiota composition within the FMT group was considerably lower than that within the control group for both FMT trials, as the FMT groups had lower dissimilarity rank values compared with the control groups (Figure 8). 

### 2.3. Composition of Microbiotas

Taxonomic analysis was conducted using the 16S rRNA gene sequencing results on the QIIME 2 platform to determine how the gut microbiota composition differed between the FMT and the control groups at the phylum and genus levels. The details are described below.

#### 2.3.1. Composition at the Phylum Level

**Figure 9:**
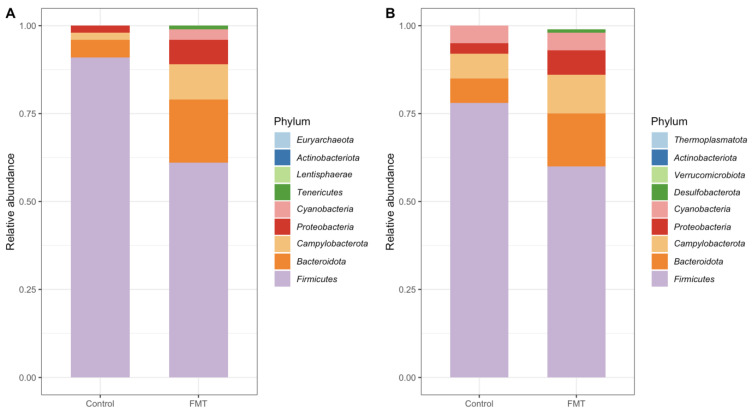
Stacked bar plot representations of the main taxa’s relative abundances at the phylum level for FMT trial 1 (**A**) and FMT trial 2 (**B**). Values represent combined data across all time points for each group/trial. The abundances of *Tenericutes* (0.37%) and *Actinobacteriota* (0.18%) in the control group were insufficient to be visually represented in Figure (**A**). Likewise, the abundances of *Actinobacteriota* (0.09%), *Desulfobacterota* (0.07%), and *Verrucomicrobiota* (0.03%) in the control group were insufficient to be visually represented in Figure (**B**).

For FMT trial 1, 9 phyla (*Firmicutes*, *Bacteroidota*, *Campylobacterota*, *Proteobacteria*, *Tenericutes*, *Actinobacteriota*, *Euryarchaeota*, *Cyanobacteria*, and *Lentisphaerae*) were detected in all samples from the FMT group, while only 6 phyla (the same ones in the FMT group, except for *Euryarchaeota*, *Cyanobacteria*, and *Lentisphaerae*) were detected in the control group in the combined dataset across all time points (Figure 9A, Table S1). Of note, among these 9 phyla, only *Euryarchaeota* is from the archaea domain; others are all bacteria. The main phylum detected from all the samples was *Firmicutes*, with a relative mean abundance of 90.63% and 60.76% in the control group and FMT group, respectively. The three next most abundant phyla were *Bacteroidota* (4.85%), *Campylobacterota* (2.16%), and *Proteobacteria* (1.80%) for the control group, and *Bacteroidota* (18.85%), *Campylobacterota* (9.89%), and *Proteobacteria* (6.67%) for the FMT group (Figure 9A). The phylum-level microbiota compositions at individual time points are shown in Figure S1. In the control group, there was an overall decline in the levels of *Firmicutes* and *Proteobacteria* as the chickens matured, while the prevalence of *Bacteriodota* and *Campylobacterota* increased (Figure S1). In the FMT group, the highest abundances of *Bacteriodota* and *Proteobacteria* were observed on DPI-5 in comparison to the other time points. In addition, there was an overall increase in *Campylobacterota* over time.

In the combined dataset across all time points for FMT trial 2, 9 phyla (*Firmicutes*, *Bacteroidota*, *Campylobacterota*, *Cyanobacteria*, *Proteobacteria*, *Actinobacteriota*, *Desulfobacterota*, *Verrucomicrobiota*, and *Thermoplasmatota*) were detected in all samples from the FMT group; the control group had 8 phyla, which included all those detected in the FMT group except for *Thermoplasmatota* (Figure 9B, Table S2). Among those 9 phyla, only *Thermoplasmatota* is from the archaea domain; others are all bacteria. The main phylum detected from all the samples was *Firmicutes*, with a relative mean abundance of 77.83% in the control group and 60.17% in the FMT groups. The three next most abundant phyla were *Bacteroidota* (7.44%), *Campylobacterota* (6.52%), and *Cyanobacteria* (5.39%) in the control group, and *Bacteroidota* (15.41%), *Campylobacterota* (10.70%), and *Proteobacteria* (6.94%) in the FMT group (Figure 9B). The phylum-level microbiota compositions at individual time points are shown in Figure S2. In the control group, there was a decline in the level of *Firmicutes* as the chickens matured, while the abundance of *Bacteroidota*, *Proteobacteria*, and *Campylobacterota* increased (Figure S2). Conversely, the abundance of *Firmicutes* gradually increased in the FMT group, whereas the abundance of *Campylobacterota* decreased over time (Figure S2).

#### 2.3.2. Composition at the Genus Level

**Figure 10:**
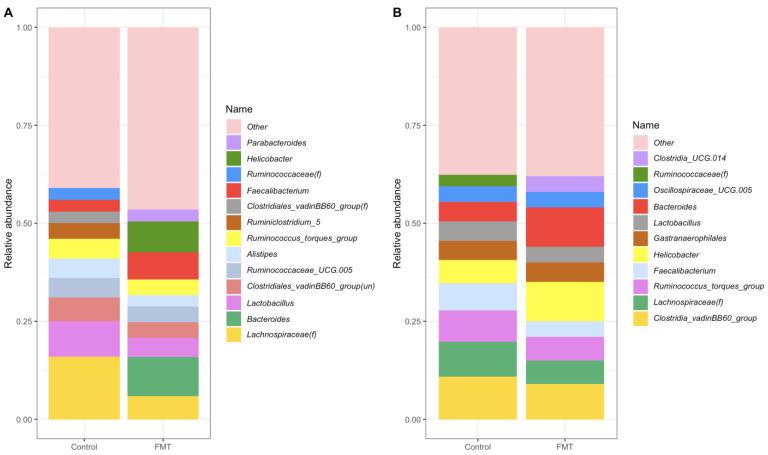
Stacked bar plot representations of the main taxa’s relative abundances at the genus level for FMT trial 1 (**A**) and FMT trial 2 (**B**). Values represent combined data across all time points for each group/trial.

The top 10 abundant genera of the control and the FMT groups for both trials are shown in Figure 10 and Tables S3 and S4. For FMT trial 1, the most abundant genera were *Bacteroides* (9.63%), *Helicobacter* (7.79%), and *Faecalibacterium* (6.60%) in the FMT group, while an unknown genus of *Lachnospiraceae(f)* (16.21%), *Lactobacillus* (8.62%), and *Clostridiales_vadinBB60_group*(un) (5.54%) were the most abundant in the control group (Figure 10A). For FMT trial 2, the most abundant genera were *Helicobacter* (10.35%), *Bacteroides* (9.60%), and *Clostridia_vadinBB60_group* (9.29%) in the FMT group, while *Clostridia_vadinBB60_group* (10.79%), *Lachnospiraceae(f)* (8.69%), and *Ruminococcus_torques_group* (7.82%) were the top three genera in the control group (Figure 10B).

#### 2.3.3. Composition of the FMT Inoculum

DNA extractions from the cecal samples used to prepare the FMT inoculum were also sequenced on the Illumina MiSeq platform under the same protocols mentioned previously. *Firmicutes* made up most (80.25%) of the bacterial phyla in the FMT inoculum, followed by *Bacteroidota* (14.702%) and *Cyanobacteria* (2.769%) (Table S5). The top five abundant genera of the FMT inoculum were *Faecalibacterium* (10.15%), *Lachnospiraceae(f)* (9.11%), *Bacteroides* (8.82%), *Lactobacillus* (7.47%), and *Phascolarctobacterium* (6.81%) (Table S6). FMT appeared to directly influence the microbial community composition and/or structure of the recipient birds in both FMT trials. For example, certain phyla present in the FMT groups only but not in the control groups (i.e., *Euryarchaeota* and *Cyanobacteria* for FMT trial 1 and *Thermoplasmatota* for FMT trial 2) (Figure 9) were also all present in the FMT inoculum (Table S5), indicating that these phyla in the FMT groups directly originated from the FMT inoculum.

### 2.4. FMT Resulted in Significant Changes in the Cecal Microbiota Composition as Revealed by ANCOM Analysis

To identify the discriminating members of the bacterial taxa (at the phylum and genus levels) in the microbiota compositions between the FMT group and the control group, ANCOM was performed. For FMT trial 1, three out of the nine identified bacterial phyla were significantly different between the control group and the FMT group. *Actinobacteriota and Firmicutes* decreased in abundance, whereas *Cyanobacteria* increased in abundance following the FMT (Table S7). Eleven percent (17/150) of the identified bacterial genera significantly differed between the control group and the FMT group in FMT trial 1 (Table 1). Whereas *Staphylococcus*, *Weissella*, *Romboutsia*, and *Proteus* decreased in abundance, *Butyricimonas, Parabacteroides*, *Parasutterella*, and *Bilophila* increased in abundance following the FMT (Table 1). 

For FMT trial 2, three out of the nine identified bacteria phyla were significantly different between the control and the FMT groups. While *Actinobacteriota* and *Firmicutes* decreased in abundance, *Desulfobacterota* increased in abundance following the FMT (Table S8). Nine percent (12/132) of the identified bacterial genera significantly differed between the control and FMT samples (Table 2). *Dorea*, *LachnospiraceaeFE2018group*, and *Proteus* decreased in abundance, whereas *Butyricimonas*, *Parabacteroides*, *Parasutterella*, and *Bilophila* increased in abundance following FMT (Table 2). It is noteworthy that *Butyricimonas*, *Parabacteroides*, *Bilophila*, *Parasutterella*, *Phascolarctobacterium*, *Fournierella*, and *Helicobacter* were all increased in the FMT groups in both trials, while *Proteus* was decreased following the FMT in both trials (Table 1 and Table 2).

### 2.5. Correlations between the Core Cecal Microbial Genera

**Figure 11:**
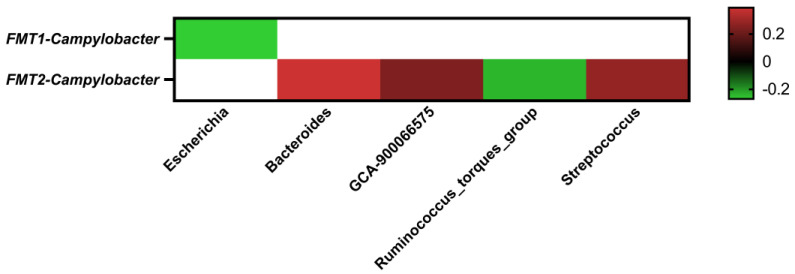
Heat map showing Pearson’s correlation coefficients for *Campylobacter* abundance and other abundant genera abundances at the genus level for both FMT trials. Not applicable is shown in white. Coefficients range from −1 to 1, with 1 representing the highest positive correlation and −1 representing the highest negative correlation. Significant correlations are colored in red (positive) or green (negative) hues. Correlations that were not significant are not shown in the plot.

The interaction among abundant genera from all the samples of FMT trial 1 and FMT trial 2 was further investigated through Pearson’s correlation analysis (Figure 11). In FMT trial 1, even though no positive correlations were found, *Campylobacter* abundance was negatively correlated with *Escherichia* (correlation coefficient = −0.27; *p*-value < 0.05). In FMT trial 2, while *Campylobacter* abundance was positively correlated with *Bacteroides* (correlation coefficient = 0.39; *p*-value < 0.05), *Lachnospiraceae*_GCA-900066575 (correlation coefficient = 0.24; *p*-value < 0.05), and *Streptococcus* (correlation coefficient = 0.28; *p*-value < 0.05), it was negatively correlated with *Ruminococcus_torques_group* (correlation coefficient = −0.24; *p*-value < 0.05).

## 3. Discussion

In the current study, we evaluated the effect of cecal microbiota derived from *Campylobacter*-free adult commercial broilers on *Campylobacter* colonization in young broilers by conducting two different FMT experiments using either a direct challenge model or a horizontal transmission model. Interestingly, we found that the FMT (performed on the day of the hatch) significantly reduced *Campylobacter* colonization in the former model (when individual birds were each orally inoculated with *C. jejuni*); however, it had no obvious effect in the latter model (when *C. jejuni*-colonized seeder birds were used to infect uninoculated birds). Previously, Gilroy et al. reported that FMT performed on the day of the hatch reduced *Campylobacter* colonization in the ceca of commercial broilers on DPI-04 shortly after direct infection via oral inoculation; however, there was no significant difference between the FMT population and the control population on DPI-10 [39]. In the present study, we observed that the FMT reduced *Campylobacter* colonization of the ceca throughout the entire study (on DPI-5, DPI-10, and DPI-15) in a similar challenge model. Gilroy et al. also reported that the FMT significantly reduced *C. jejuni* colonization in the intestine of contact birds in the seeder bird infection model throughout (up to DPI-14, DPI-12) two separate experiments [39]. In contrast, we did not observe any significant inhibitory effect of the FMT on *C. jejuni* colonization in the ceca of co-mingled birds in the seeder bird infection model conducted in the present study, although the FMT materials used in both trials were from the same preparation. Even though the exact reason behind the discrepant findings between the two studies conducted by Gilroy et al. and us is difficult to determine definitively, potential differences in certain characteristics of the chickens (including the birds used for FMT preparation (commercial broilers in our study vs. laboratory-raised broilers in Gilroy et al. [39]), recipients of the FMT, and seeder birds), the FMTs, the *C. jejuni* strains, and the experimental designs may have contributed to the contrasting outcomes observed. It should be noted that it is difficult to assess the exact biological significance and impact of the colonization reduction (~log_10_ 1.2–2. 54 CFU/g feces) seen in FMT trial 1 in this study; however, previous studies indicated that a 1- to 3-log_10_ reduction in *Campylobacter* loads at preharvest level (broiler ceca) would translate to significant reductions in postharvest (carcass) contamination and subsequent zoonotic transmission risk [46,47]. 

The exact reason for the differences between the direct infection model and the seeder bird infection model in the reduction of *Campylobacter* colonization in the intestine of broilers following FMT (Figure 1) is unknown but could be explained by several possibilities. First, in the seeder bird infection model, *Campylobacter* had adapted in the intestine of seeder birds prior to its spread to the co-mingled birds. The intestinal adaption stage could enhance the organism’s ability to transmit horizontally and/or colonize chickens, overriding the protective effect of intestinal microbiota. On the contrary, *Campylobacter* grown from an in vitro culture was given to each bird directly by oral gavage in the direct inoculation model, where the organism was not initially adapted to the gastrointestinal environment, which could affect its ability to colonize the intestinal tract. Second, in the seeder bird model, it is possible that the unchallenged contact birds were exposed to a high level of *Campylobacter* shed in the droppings of the seeders continuously for a prolonged period throughout the experiment. The seeder birds did not receive FMT and were well-colonized with *Campylobacter* at high levels. Thus, repeated exposure via coprophagy to a higher level of well-adapted *Campylobacter* from the seeder birds may have overwhelmed the rather weak protective effect of the FMT that was otherwise observed in the direct challenge model. 

In addition to reducing *Campylobacter* colonization, the FMT also altered the cecal microbiota composition and structure significantly in young broiler chickens. We elected to focus on the cecal microbiota since the cecum is an important site for *Campylobacter* colonization, and cecal microbiota also plays an essential role in host immunity [48]. For the within-group microbial diversity (i.e., alpha diversity), the FMT increased the cecal microbiota species richness and phylogenetic diversity (Figure 2, Figure 3, Figure 4 and Figure 5). In a previous study, Gilroy et al. found that even though FMT increased the phylogenetic diversity of the cecal microbiota, it did not have any significant effect on the bacterial species’ richness [39]. When examining the alpha diversity for the samples grouped by treatment status and sampling time (Figure 3 and Figure 5), an increasing trend of species richness and phylogenetic diversity was seen in the control groups, while the FMT groups had high levels of both indexes from the beginning. This difference in the microbiota dynamics between the groups was expected since the birds in the FMT groups were inoculated with the microbiota from adult broilers. Interestingly, the Pielou’s evenness diversity between the two groups was not significantly different at most of the sampling points, even though the Shannon index differed significantly between the groups. It is likely that the higher value of the Shannon index of the FMT groups was mainly due to the greater species richness found in the FMT groups, as the Shannon index is calculated by both species diversity and evenness within a sample. The finding of comparable levels of microbial evenness between the groups was not unexpected, as the control birds could have well-balanced gut microbiota with comparable evenness to the FMT group under normal experimental conditions without any additional interventions. It is worth noting that the overall alpha diversity patterns observed in both FMT and the control groups between the two FMT trials were highly comparable, suggesting that alpha diversity did not appear to be a significant contributor to the discrepant effect of FMT in *Campylobacter* colonization between the two FMT trials.

Aside from the alpha diversity differences, variations in the gut microbiota community structure (i.e., beta diversity) between the FMT population and the control population were also observed (Figure 6 and Figure 7). In the study of Gilroy et al., the cecal microbiota composition of the FMT population showed a significant separation from the control populations, and this was somewhat consistent with our findings [39]. In both of our FMT trials, the FMT and control populations showed unique clusters and a clear separation from each other. The ANOSIM test results further confirmed that the FMT treatment resulted in significant taxonomic composition differences between the treatment groups in both FMT trials (Figure 8). Moreover, we observed in both trials that while the control groups had obvious separations of the microbiota compositions by sampling time, the microbiota compositions of the FMT groups tended to cluster together irrespective of the sampling time (Figure 6B and Figure 7B). This finding was not unexpected since the birds in the control groups did not receive any external microbiota; their gut microbiota developed gradually over time and exhibited distinct compositions as the experiment progressed. On the contrary, as the chickens in the FMT groups received mature fecal microbiota on the day of the hatch, their gut microbiota compositions were already shaped (largely by the FMT inoculum) by the time of the first sampling (around two weeks after the FMT) and thus did not undergo such dramatic changes over time during the rest of the experiment.

Consistent with other studies on the chicken gut microbiome [49,50], *Firmicutes* and *Bacteroidota* were the major bacterial phyla identified in the cecal microbiotas in both FMT trials conducted in the present study (Figure 9), as were in the FMT inoculum (Table S1). It is interesting to note that while the abundance of *Firmicutes* decreased significantly, a reverse trend was observed for *Bacteroidota* following the administration of FMT in both trials. Of note, several core genera found in both FMT trials, including *Lactobacillus*, *Faecalibacterium*, *Ruminococcaceae_UCG.005*, and *Clostridia_vadinBB60_group* (Figure 10), are all members of the *Firmicutes* phylum. *Bacteroidota* is mainly composed of Gram-negative bacteria, and some gastrointestinal *Bacteroidota* species produce short-chain fatty acids (SCFA) as major end-products in the gut. In this study, *Bacteroidota* was mainly represented by the genera *Bacteroides*, *Alistipes*, and *Parabacteroides*. *Campylobacterota* and *Proteobacteria* were the next two most abundant phyla identified in both FMT trials in the current study and are also commonly found in the chicken cecum [50,51]. *Helicobacter* and *Campylobacter* were the main genera representing *Campylobacterota*, while *Parasutterella* and *Escherichia* were the main genera representing *Proteobacteria* in both FMT trials. It was observed that while the abundance of *Actinobacteriota* was decreased in both trials following the FMT, those of *Cyanobacteria* and *Desulfobacterota* were increased after the FMT in either one of the trials (Tables S3 and S4). Of note, all three phyla were also found in the FMT inoculum (Table S1). 

Several genera, such as *Butyricimonas, Parabacteroides, Parasutterella, Bilophila, Phascolarctobacterium, Fournierella,* and *Helicobacter,* were consistently increased in both FMT trials (Table 2 and Table 3) and were also found in the FMT inoculum (partly shown in Table S2). The observed elevation of these genera following FMT suggests their potential involvement in shaping the microbiota composition towards a state that might be less conducive to *Campylobacter* colonization in the chicken gut. Also, their presence in the FMT inoculum further supports a potential role in driving the observed effects. The consistent outcomes in both FMT experiments lend strength to the notion that these microbial communities could influence the colonization dynamics of *Campylobacter* in broilers. The increased abundance of genera like *Butyricimonas*, *Parabacteroides*, and *Phascolarctobacterium*, which are associated with the production of SCFAs and fermentation of dietary fiber, could indicate a potential shift towards a more favorable microbial ecosystem [52,53,54]. SCFAs are known to contribute to gut health by promoting the growth of beneficial microorganisms and creating an environment less hospitable to pathogens [55]. On the other hand, the increased abundance of genera such as *Bilophila* and *Helicobacter* may warrant further investigation for evaluation of their implications for *Campylobacter* colonization and gut health.

In addition to the ANCOM results, the correlation coefficients also revealed several genera that were significantly positively or negatively correlated with the abundance of *Campylobacter* (Figure 11). It is worth mentioning that *Escherichia* was the only identified genus that was negatively correlated with *Campylobacter* abundance in FMT trial 1, in which the FMT showed a significant inhibitory effect on the *Campylobacter* colonization in the recipient birds throughout the trial (Figure 1A). It was reported that giving *E. coli* Nissle 1917 supplements, either in the drinking water or orally, resulted in at least a 2.0 log reduction of *C. jejuni* colonization in chickens [56]. It is also worth noting that no such correlation for the *E. coli* was observed in FMT trial 2, where the FMT had no inhibitory effect on *Campylobacter* colonization (Figure 1B). For FMT trial 2, *Ruminococcus_torques_group* was the only identified genus that was significantly negatively correlated with *Campylobacter* abundance. Interestingly, in a search for potential gut bacteria related to improvements in bird performance, as measured by feed efficiency, both *E. coli* and *Ruminococcus_torques* were identified to be potentially performance-related phylotypes in the gut microbiota of broiler chickens [57]. *Bacteriodes* was found to be the genus that has the highest significant positive correlation coefficients with *Campylobacter* abundance in the current study. This result is consistent with our previous observation, where *Bacteriodes* was identified as the most abundant genus in the cecal microbiota of *Campylobacter*-positive commercial broilers [45]. *Streptococcus* was also identified to be significantly positively correlated with *Campylobacter* abundance in our analysis, which is in agreement with a recent study conducted on commercial broiler chickens in Belgium where *Streptococcus* was reported to be more abundant in *Campylobacter*-colonized flocks [58].

Presently, the majority of FMT experiments utilize donor feces from humans or animals to create the FMT inoculum. However, there are multiple concerns regarding the inclusion of unnecessary components in the inoculum. During FMT trial 2, we observed a reduction in body weight among the birds that received the FMT compared with the control group (results not shown), suggesting that the birds may have been inadvertently affected by the FMT inoculum. It is possible that some undefined components in the FMT inoculum, which was crude and directly from commercial birds, might have contributed to the weight gain reduction. Thus, synthetic gut microbiota or defined microbiome consortia are better options for microbiota-based interventions [59,60,61,62]. In the future, it would be interesting to evaluate synthetic FMT (sFMT) materials comprising the microbial taxa found to be negatively correlated with *Campylobacter* colonization as microbiota-based interventions for the preharvest control of *Campylobacter* in poultry. It should be pointed out that a single probiotic may not yield consistent efficacy under different conditions; therefore, a microbiota consortium is expected to provide better outcomes regarding *Campylobacter* mitigation in poultry. Even though formulating an effective microbiota consortium could prove to be a challenging task, this is likely to be improved by continuing to perform well-designed FMT studies under different conditions.

## 4. Materials and Methods

### 4.1. Bacterial Strains and Culture Conditions

The *Campylobacter jejuni* W7 strain (a highly motile variant of NCTC 11168) was routinely cultured on Mueller–Hinton (MH) agar (Becton, Dickinson and Company, Sparks, MD, USA) at 42 °C in microaerobic conditions (5% O_2_, 10% CO_2_, 85% N_2_) [63]. The MH agar was supplemented with the *Campylobacter* growth supplement (SR084E; Oxoid, Basingstoke, UK) and modified Preston *Campylobacter* selective supplement (SR0204E; Oxoid) for isolation of the organism from fecal samples as needed. Before the animal study, the motility of the isolate was confirmed using a 0.4% Bacto Agar (Becton, Dickinson and Company, Sparks, MD, USA) MH plate as described previously [63].

### 4.2. Preparation of Fecal Transplantation Material

The cecal contents of adult, *Campylobacter*-free commercial broiler chickens were used to prepare the fecal transplantation material employed in this study. The samples were sourced from a conventional broiler farm that was previously found to be consistently free of *Campylobacter* over multiple production cycles, as determined in our previous study [45]. Sample collection was completed by the company personnel when the birds were still on the farm at 5 weeks of age. For this purpose, a cohort of 30 randomly picked birds were humanely euthanized via cervical dislocation following the company’s protocols. The entire ceca of each bird was individually collected in sterile Whirlpak bags and then placed into Ziploc bags in groups of five, placed on ice, and kept refrigerated until the shipment. Subsequently, the samples were shipped overnight to Iowa State University (ISU) in insulated boxes with ice packs to maintain optimal conditions. On the same day of arrival at the laboratory, halves of the cecal contents were used for the *Campylobacter* culture and microbiota analysis, as described previously [45]. The other halves of the cecal contents were collected under anaerobic conditions, pooled together, and diluted 1:20 in sterile, reduced Phosphate Buffer Solution (PBS + 10% glycerol) to prepare the fecal transplantation material, as described in a previous study [64]. The fecal transplantation material was saved in multiple 50 mL sterile polypropylene centrifuge tubes (Corning, Glendale, AZ, USA) at −80 °C until further use. The fecal material was thawed at 4 °C overnight before the transplantation experiments described below. It is worth mentioning that each tube containing the fecal material was used only once (i.e., one tube per study). 

### 4.3. Fecal Microbiota Transplantation (FMT) Trials

All the procedures and protocols performed in the animal experiments were approved by the ISU Institutional Animal Care and Use Committee (IACUC-21-266) prior to the commencement of the study. Two FMT trials were performed to assess FMT’s effect on *Campylobacter* colonization in young birds by employing either a direct challenge or seeder bird challenge model, as described below. In both trials, newly hatched broiler chicks of the same breed were obtained from the same commercial hatchery. Upon arrival at the ISU animal facility, feed (standard, antibiotic-free, commercial broiler starter feed) was withdrawn until the FMT material was given while water was provided ad libitum. The FMT material was given within a few hours of the arrival via oral gavage (100 µL/bird). Of note, chicks in the control groups were given 100 µL sterile PBS (containing 10% glycerol) instead of the FMT material. FMT was performed similarly in both trials, and the major procedures are summarized in Table 3.

In FMT trial 1, sixty 1-day-old broiler chicks were randomly assigned into two groups (FMT and control; 30 birds per group) at the ISU animal facility. The birds were housed in floor pens with fresh wood shavings, with enough separation between the pens to minimize cross-contamination. The FMT was performed as described above. Cloacal swabs were collected when the birds were 13 days old and were cultured on the selective MH agar; all the birds were confirmed to be *Campylobacter*-free. At 15 days of age, six chicks from each group were euthanized for collection of the cecal contents to serve as the microbiota baseline samples before *C. jejuni* inoculation. All remaining birds (24 in each group) were inoculated with ~1 × 10^6^ colony-forming units (CFU)/bird of the *C. jejuni* W7 strain via oral gavage. The bacterial inoculum was prepared from the fresh log-phase growth on MH agar and by re-suspending it in sterile PBS to adjust OD_600_ to the desired level; each bird received 200 µL of this suspension. The actual concentration of the inoculum (CFU/mL) was also determined by viable plate counting. Eight birds from each group were euthanized on days post inoculation DPI-5, DPI-10, and DPI-15. At necropsy, the cecal contents were collected in sterile tubes on ice and processed for the quantitative *Campylobacter* culture (using serially diluted samples) in each bird using the selective MH agar plates. In addition, cecal contents were collected in ZR BashingBead™ lysis tubes (Zymo Research, Irvine, CA, USA) on ice during the necropsy and processed for DNA isolation and microbiota analysis, as described below. The agar plates were incubated at 42 °C microaerobically for 48 h for enumeration of *Campylobacter*-like colonies. The identity of the isolates (typically one isolate per positive bird) was confirmed by MALDI-TOF MS following the SOPs in place at the Veterinary Diagnostic Laboratory of ISU. 

In FMT trial 2, of the eighty-eight 1-day-old broiler chicks delivered to the ISU animal facility, eight were randomly selected and placed in a separate group to serve as the seeder birds, which had immediate access to the standard feed (the same type used in trial 1) and water. Of note, the seeder birds did not receive any FMT. The remaining eighty 1-day-old chicks were evenly divided into two groups (FMT and control) and housed in separate floor pens with wood shavings. The FMT was performed as described above. At 5 days of age, the seeder birds were confirmed to be *Campylobacter*-free by culturing the cloacal swabs on the selective MH agar plates. At 7 days of age, all the seeder birds were orally inoculated with the same *C. jejuni* W7 strain used in trial 1 (each bird received ~1 × 10^7^ CFU in 200 µL total volume of inoculum). At 12 days of age, cloacal swabs from all birds in the FMT group, the control group, and the seeder birds were taken to determine their *Campylobacter* status; it was confirmed that while the FMT and control groups were *Campylobacter*-negative, the seeder birds were all colonized. At 14 days of age, 10 birds from the FMT group and 10 birds from the control group were euthanized, and the cecal contents were collected to serve as the microbiota baseline samples. On the same day, the seeder birds were transferred to the FMT group and the control group (3 seeders per group) and allowed to co-mingle with the reminder of birds (n = 30) in each group. Eleven birds (ten contact birds and one seeder bird) from each group were euthanized on days post-mingling DPM-5, DPM-10, and DPM-15. At necropsy, the cecal contents of the contact birds were collected for the quantitative *Campylobacter* culture and microbiota analysis, as described in FMT trial 1 above. 

### 4.4. DNA Extraction and 16S rRNA Gene Sequencing

Briefly, within a couple of hours of sample collection at necropsy, 200 mg of feces from each cecum was transferred to a 2 mL ZR BashingBead™ lysis tube and mixed with 250 µL of deionized sterile water, 750 µL of lysis solution (included in the kit), and 50 µL of (from 20 mg/mL stock) proteinase K (Qiagen, Germantown, MD, USA). The samples underwent processing using a bead beater (Bullet Blender, Next Advance, NY, USA) for 10 min, followed by incubation at a minimum of 30 min at 55 °C. The lysis tubes were then subjected to microcentrifugation at 10,000× *g* for 3 min, and the resultant supernatant was collected and transferred to the columns. Subsequently, DNA Wash Buffers 1 and 2 were used for washing, and the final product was eluted using DNase/RNase-free water. The concentration of eluted DNA was measured initially using a NanoDrop 3300 Fluorospectrophotometer (NanoDrop Technologies, Wilmington, DE, USA) and later validated by a Qubit fluorometer (Turner BioSystems, Sunnyvale, CA, USA). Further, 16S rRNA gene amplicon sequencing and analysis were conducted to determine the effects of FMT on gut microbiota. DNA extractions from the cecal samples were performed following the ZymoBIOMICS™ protocol (Zymo Research). The V4 hypervariable regions of the bacterial 16S rRNA gene were amplified using a universal 16S forward primer (515F: GTGYCAGCMGCCGCGGTAA) and a reverse primer (806R: GGACTACNVGGGTWTCTAAT). Following normalization, all the DNA extracts were transferred to 96-well plates and submitted for library preparation and sequencing at the DNA Facility of ISU. The Earth Microbiome Project protocol was followed for sequencing on the Illumina MiSeq platform (Company info) with 2 × 250 paired-end technology in a single flow cell lane [65]. 

### 4.5. Bioinformatics and Statistical Analysis

Bioinformatic analyses of the 16S rRNA sequence data were performed using the QIIME 2 (version 2021.04) pipeline [66]. Specifically, the sequencing data obtained from the ISU DNA facility underwent demultiplexing, followed by denoising using the DADA2 method to eliminate noisy sequences, remove chimeric sequences, and cluster similar sequences into amplicon sequence variants (ASVs) [67]. The taxonomy classification was completed by comparing the ASVs to the SILVA 16S rRNA gene database [68]. Alpha and beta diversity metrics were computed to evaluate the effects of FMT on the richness and diversity of gut microbiota, as described previously [45]. Briefly, for the alpha diversity, the richness was estimated using the observed features, and the evenness was determined using the Shannon index and Pielou’s evenness index. The phylogenetic diversity was evaluated by Faith’s phylogenetic diversity index. The beta diversity analysis was measured by weighted UniFrac distances (a quantitative measure of community dissimilarity that incorporates the presence/absence of microbial taxa, their relative abundances, and phylogenetic relationships between the taxa) and illustrated in a two-dimensional principal coordinate analysis (PCoA) plot. Analysis of similarity (ANOSIM) was computed utilizing the vegan package in the R programming language [69]. Differentially abundant phyla and genera between the FMT group and the control group were determined by analysis of the compositions of microbiomes (ANCOM) [70]. Bioinformatics analysis information can be found at the link: https://github.com/JinjiPang/FMT-MDPI (accessed on 8 March 2023).

*Campylobacter* counts from the cecal samples were expressed as log_10_ CFU/g feces. Non-parametric Wilcoxon and Kruskal–Wallis tests were performed to compare the *Campylobacter* colonization levels among different groups and time points. A *p*-value of <0.05 is considered significant for all analyses. Pearson’s correlation coefficient was used to test the correlations between *Campylobacter* abundance and the dominant genera of microbiota.

## 5. Conclusions

The key finding of the current study is that early (performed on the day of the hatch) FMT using the cecal contents of *Campylobacter*-free adult commercial broilers significantly reduced *Campylobacter* colonization in young broilers when the *Campylobacter* challenge was given to individual birds via oral gavage; however, not when the challenge was conducted horizontally by use of seeder birds. Furthermore, it was shown that the FMT significantly affected the subsequent temporal development of the gut microbiota in the recipient birds. Microbial diversity and composition differed significantly between the control and FMT groups. Certain taxa (e.g., *Escherichia*, *Ruminococcus_torques-group*) were found to be negatively correlated with *Campylobacter* abundance in the ceca and thus could be potential targets for the development of microbiota-based *Campylobacter* control measures on poultry farms. However, the key mechanisms involved in the reduction of *Campylobacter* colonization in broiler chickens after fecal microbiota transplantation still remain to be identified. Future studies are needed to determine the exact role of cecal microbiota on *Campylobacter’s* growth and colonization in the broiler intestinal tract and develop FMT materials that may be used practically in commercial settings.

## Figures and Tables

**Table 1:** Analysis of composition of microbiomes (ANCOM) test results for differentially abundant genera between the FMT group and the control group for FMT trial 1.

Genus	Abundances 50%	Abundances 50%	W *	Change
	Control	FMT		FMT vs. Control
*Staphylococcus*	1184	7	148	Decreased
*Weissella*	105.5	4.5	144	Decreased
*Romboutsia*	1401	67.5	144	Decreased
*Proteus*	36.5	1	144	Decreased
*Clostridium_innocuum_group*	25.5	1	142	Decreased
*Brachybacterium*	16	1	138	Decreased
*CHKCI002*	79	11	133	Decreased
*Bacteroides*	1	7528.5	148	Increased
*Butyricimonas*	1	947	146	Increased
*Parabacteroides*	1	2487.5	146	Increased
*Parasutterella*	1	2173	145	Increased
*uncultured_Clostridia_bacterium*	1	622.5	144	Increased
*Bilophila*	1	564.5	144	Increased
*Fournierella*	1	207.5	141	Increased
*Odoribacter*	1	134	140	Increased
*Helicobacter*	9	5963.5	140	Increased
*Phascolarctobacterium*	1	134	139	Increased

* High W values indicate significant differences in abundance levels between the groups. The higher the W value, the greater the differences in abundance levels between groups. Fifty percentile abundances of features by the group are also listed. The higher the value, the more abundant the taxon.

**Table 2:** Analysis of composition of microbiomes (ANCOM) test results for differentially abundant genera between the FMT group and the control group for FMT trial 2.

Genus	Abundances 50%	Abundances 50%	W *	Change
	Control	FMT		FMT vs. Control
*Dorea*	770	1	113	Decreased
*Proteus*	4	1	113	Decreased
*LachnospiraceaeFE2018group*	12	1	110	Decreased
*Butyricimonas*	1	600	130	Increased
*Parabacteroides*	1	1366.5	130	Increased
*Bilophila*	1	609.5	130	Increased
*Parasutterella*	1	1421.5	130	Increased
*Phascolarctobacterium*	1	46.5	124	Increased
*Pygmaiobacter*	1	42.5	110	Increased
*Ruminococcus*	97	639.5	104	Increased
*Fournierella*	1	41.5	102	Increased
*Helicobacter*	667	4503	98	Increased

* High W values indicate significant differences in abundance levels between groups. The higher the W value, the greater the differences in abundance levels between groups. Fifty percentile abundances of features by the group are also listed. The higher the value, the more abundant the taxon.

**Table 3:** Summary of the major activities performed in the current study.

Trial	Group	FMT	Cloacal Swab	*C. jejuni* Inoculation	Necropsy
FMT trial 1	FMT	Yes	Yes	Oral gavage	DPI-0, 5, 10, 15
	Control	No	Yes	Oral gavage	DPI-0, 5, 10, 15
FMT trial 2	FMT	Yes	Yes	Seeder birds	DPM-0, 5, 10, 15
	Control	No	Yes	Seeder birds	DPM-0, 5, 10, 15

The birds (commercial broilers) in the FMT groups were given the FMT inoculum on the day of hatch, while the birds in the control groups were given the sham inoculum at the same time. Cloacal swabs were taken from the birds before *Campylobacter* infection to confirm their *Campylobacter*-free status. The birds were infected with *C. jejuni* by either oral gavage (FMT trial 1) or co-mingling with *Campylobacter*-colonized seeder birds (FMT trial 2) approximately two weeks after the FMT. Necropsies were performed on DPI-0, DPI-5, DPI-10, and DPI-15 in FMT trial 1 (8 birds each time) and on DPM-0, DPM-5, DPM-10, and DPM-15 in FMT trial 2 (10 birds each time). Cecal contents were collected at necropsy and processed for quantitative *Campylobacter* culture and 16S rRNA gene sequencing-based microbiota analysis.

## Data Availability

Data are available upon request. The 16S rRNA sequence data from both FMT trials were submitted to the NCBI SRA database under BioProject accession no.PRJNA1019766.

## Supplementary Materials

The following supporting information can be downloaded at: https://www.mdpi.com/article/10.3390/antibiotics12101503/s1, Figure S1: Stacked bar plot representations of the main taxa’s relative abundances at the phylum level for FMT trial 1, grouped by treatment status (C: Control; T: FMT) and sampling time (DPI-0, DPI-5, DPI-10, and DPI-15); Figure S2: Stacked bar plot representations of the main taxa’s relative abundances at the phylum level for FMT trial 2, grouped by treatment status (C: Control; T: FMT) and sampling time (DPM-0, DPM-5, DPM-10, and DPM-15); Table S1: Taxonomic relative abundances of the FMT inoculum at the phylum level for FMT trial 1.; Table S2: Taxonomic relative abundances of the control and FMT groups at the phylum level for FMT trial 2; Table S3: Top ten abundant genera of the control and FMT groups for FMT trial 1; Table S4: Top ten abundant genera of the control and FMT groups for FMT trial 2; Table S5: Taxonomic relative abundances of the FMT inoculum at the phylum level; Table S6: Taxonomic relative abundances of the FMT inoculum at the genus level; Table S7: ANCOM test results for differentially abundant phyla between the FMT group and the control group for FMT trial 1; Table S8: ANCOM test results for differentially abundant phyla between the FMT group and the control group for FMT trial 2.
