# Supplementary material for: Fecal Microbiota Transplantation Reduces Campylobacter jejuni Colonization in Young Broiler Chickens Challenged by Oral Gavage but Not by Seeder Birds
Source: Antibiotics (Basel). 2023 Oct 2;12(10):1503. doi: 10.3390/antibiotics12101503 (PMC10604036; doi:10.3390/antibiotics12101503)
Supplement: Supplementary file 1 [file antibiotics-12-01503-s001.zip › antibiotics-2621304-supplementary.pdf]

Supplementary materials

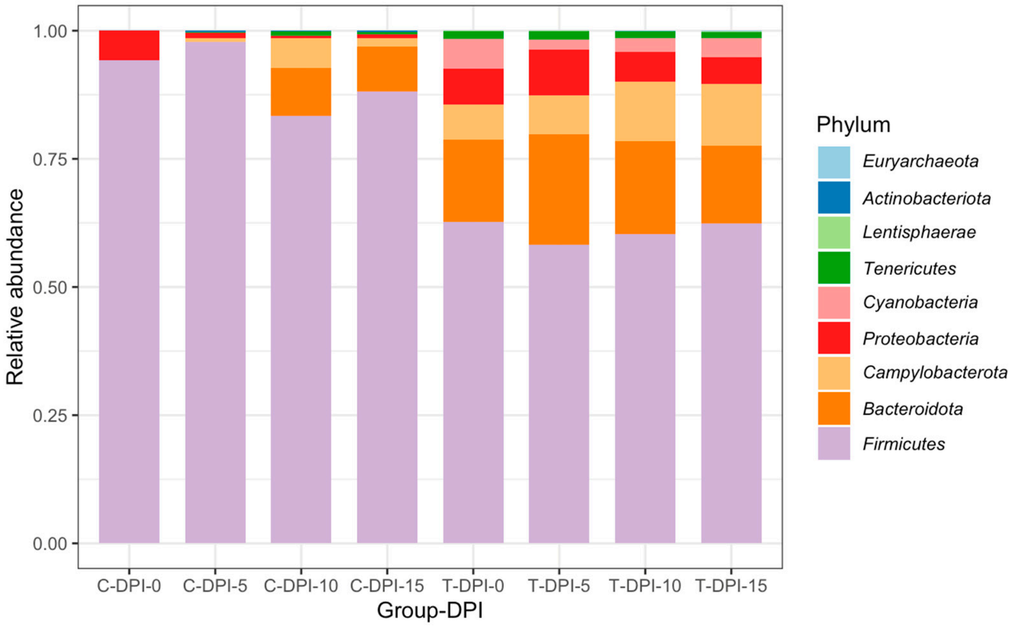

Figure S1. Stacked bar-plot representations of the main taxa's relative abundances at the phylum level for FMT trial 1, grouped by treatment status (C: Control; T: FMT) and sampling time (DPI-0, 5, 10, 15).

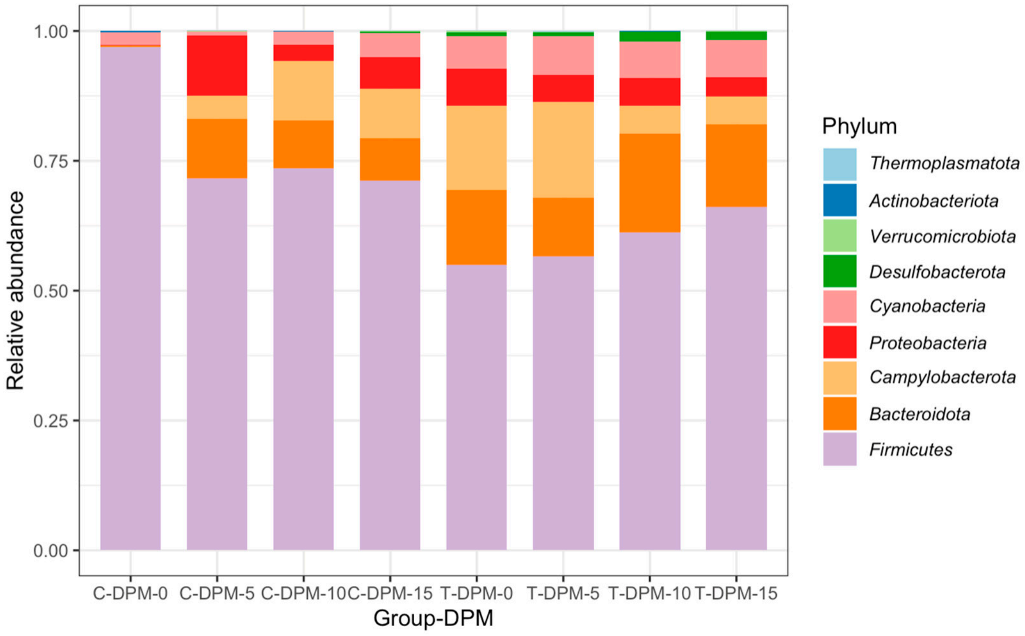

Figure S2. Stacked bar-plot representations of the main taxa's relative abundances at the phylum level for FMT trial 2, grouped by treatment status (C: Control; T: FMT) and sampling time (DPM-0, 5, 10, 15).

Table S1. Taxonomic relative abundances of the control and FMT groups at the phylum level for FMT trial 1.

Phylum	Control	FMT
Firmicutes	90.630%	60.760%
Bacteroidota	4.849%	17.850%
Campylobacterota	2.162%	9.891%
Proteobacteria	1.796%	6.571%
Tenericutes	0.378%	1.385%
Actinobacteriota	0.183%	0.014%
Euryarchaeota	0.000%	0.071%
Cyanobacteria	0.000%	3.393%
Lentisphaerae	0.000%	0.067%

Table S2. Taxonomic relative abundances of the control and FMT groups at the phylum level for FMT trial 2.

Phylum	Control	FMT
Firmicutes	77.835%	60.172%
Bacteroidota	7.442%	15.409%
Campylobacterota	6.524%	10.704%
Cyanobacteria	5.386%	5.261%
Proteobacteria	2.621%	6.936%
Desulfobacterota	0.067%	1.358%
Verrucomicrobiota	0.031%	0.108%
Actinobacteriota	0.094%	0.024%
Thermoplasmatota	0.000%	0.028%

Table S3. Top ten abundant genera of the control and FMT groups for FMT trial 1.

Rank	Control	FMT
1	*Lachnospiraceae(f)-16.21%*	*Bacteroides-9.63%*
2	*Lactobacillus-8.62%*	*Helicobacter-7.79%*
3	*Clostridiales_vadinBB60_group(un)-5.54%*	*Faecalibacterium-6.60%*
4	*Ruminococcaceae_UCG.005-5.44%*	*Lachnospiraceae(f)-6.36%*
5	*Alistipes-4.84%*	*Lactobacillus-4.59%*
6	*Ruminococcus.torques_group-4.50%*	*Clostridiales_vadinBB60_group(un)-4.32%*
7	*Ruminiclostridium_5-4.40%*	*Ruminococcaceae_UCG.005-3.79%*
8	*Clostridiales_vadinBB60_group(f)-3.38%*	*Ruminococcus.torques_group-3.55%*
9	*Faecalibacterium-3.25%*	*Alistipes-3.38%*
10	*Ruminococcaceae(f)-2.86%*	*Parabacteroides-3.34%*

Table S4. Top ten abundant genera of the control and FMT groups for FMT trial 2.

Rank	Control	FMT
1	*Clostridia_vadinBB60_group-10.79%*	*Helicobacter -10.35%*
2	*Lachnospiraceae(f)-8.69%*	*Bacteroides -9.60%*
3	*Ruminococcus.torques_group-7.82%*	*Clostridia_vadinBB60_group -9.29%*
4	*Faecalibacterium-6.53%*	*Ruminococcus._torques_group -6.20%*
5	*Helicobacter-5.79%*	*Lachnospiraceae(f)-5.65%*
6	*Gastranaerophilales -5.39%*	*Gastranaerophilales -5.26%*
7	*Lactobacillus -5.17%*	*UCG.005-4.26%*
8	*Bacteroides-5.12%*	*Faecalibacterium -3.94%*
9	*UCG.005-4.14%*	*Lactobacillus -3.92%*
10	*Ruminococcaceae(f)-2.71%*	*Clostridia_UCG.014-3.60%*

Table S5. Taxonomic relative abundances of the FMT inoculum at the phylum level.

Rank	Phylum	Relative abundance
1	*Firmicutes*	80.255%
2	*Bacteroidota*	14.702%
3	*Cyanobacteria*	2.769%
4	*Proteobacteria*	0.975%
5	*Actinobacteriota*	0.734%
6	*Desulfobacterota*	0.415%
7	*Campylobacterota*	0.084%
8	*Thermoplasmatota*	0.049%
9	*Euryarchaeota*	0.010%
10	*Verrucomicrobiota*	0.008%

Table S6. Taxonomic relative abundances of the FMT inoculum at the genus level.

Rank	Genus	Relative abundance
1	*Faecalibacterium*	10.15%
2	*Lachnospiraceae(f)*	9.11%
3	*Bacteroides*	8.82%
4	*Lactobacillus*	7.47%
5	*Phascolarctobacterium*	6.81%
6	*Ruminococcus_torques_group*	5.06%
7	*Clostridia_UCG.014*	4.02%
8	*UCG.005*	3.72%
9	*Blautia*	2.99%
10	*Subdoligranulum*	2.85%
11	*Gastranaerophilales*	2.77%
12	*Clostridia_vadinBB60_group*	2.76%
13	*Christensenellaceae_R.7_group*	2.57%
14	*Alistipes*	2.41%
15	*Oscillospiraceae(f)*	2.18%
16	Other	26.31%

Table S7. ANCOM test results for differentially abundant phyla between the FMT group and the control group for FMT trial 1.

Phylum	Abundances 50% Control	Abundances 50% FMT	W*	Change FMT vs. Control
Actinobacteriota	125.5	11.5	8	Decreased
Firmicutes	72747.5	47668.5	8	Decreased
Cyanobacteria	1.0	2167.0	8	Increased

* The ANCOM results for phyla differed significantly in abundance levels between groups. High W values indicate significant differences in abundance levels between groups. The higher the W value, the greater the differences in abundance levels between groups. Fifty percentile abundances of features by the group are also listed. The higher the value, the more abundant the taxon.

Table S8. ANCOM test results for differentially abundant phyla between the FMT group and the control group for FMT trial 2.

Phylum	Abundances 50% Control	Abundances 50% FMT	W*	Change FMT vs. Control
Actinobacteriota	20.0	11.5	8	Decreased
Firmicutes	36578.0	33204.5	8	Decreased
Desulfobacterota	1.0	650.5	8	Increased

* The ANCOM results for phyla differed significantly in abundance levels between groups. High W values indicate significant differences in abundance levels between groups. The higher the W value, the greater the differences in abundance levels between groups. Fifty percentile abundances of features by the group are also listed. The higher the value, the more abundant the taxon.
